# Establishing severe acute respiratory infection (SARI) surveillance in a sentinel hospital, Ireland, 2021 to 2022

**DOI:** 10.2807/1560-7917.ES.2023.28.23.2200740

**Published:** 2023-06-08

**Authors:** Melissa Brady, Roisin Duffy, Lisa Domegan, Abigail Salmon, Binita Maharjan, Cathal O'Broin, Charlene Bennett, James Christle, Jeff Connell, Laura Feeney, Nadra Nurdin, Patrick Mallon, Peter Doran, Rosa McNamara, Sarah O'Grady, Sinead McDermott, Naomi Petty-Saphon, Joan O’Donnell

**Affiliations:** 1 European Programme for Intervention Epidemiology Training (EPIET), European Centre for Disease Prevention and Control (ECDC), Stockholm, Sweden; 2 Health Service Executive-Health Protection Surveillance Centre (HPSC), Dublin, Ireland; 3 Department of Microbiology, St. Vincent’s Hospital, Dublin, Ireland; 4 University College Dublin (UCD) Clinical Research Centre, Dublin, Ireland; 5 Department of Infectious Diseases, St. Vincent’s Hospital, Dublin, Ireland; 6 University College Dublin (UCD) National Virus Reference Laboratory, Dublin, Ireland; 7 University College Dublin (UCD) School of Medicine, Dublin, Ireland; 8 Emergency Department, St. Vincent’s Hospital, Dublin, Ireland; 9 Department of Public Health, Eastern Region of Ireland, Dublin, Ireland; 10 University College Dublin (UCD) Centre for Experimental Pathogen Host Research, Ireland

**Keywords:** Severe Acute Respiratory Infection, Syndromic surveillance, Ireland, COVID-19, Influenza, respiratory syncytial virus, RSV

## Abstract

**Background:**

In 2020, due to the COVID-19 pandemic, the European Centre for Disease Prevention and Control (ECDC) accelerated development of European-level severe acute respiratory infection (SARI) surveillance.

**Aim:**

We aimed to establish SARI surveillance in one Irish hospital as part of a European network E-SARI-NET.

**Methods:**

We used routine emergency department records to identify cases in one adult acute hospital. The SARI case definition was adapted from the ECDC clinical criteria for a possible COVID-19 case. Clinical data were collected using an online questionnaire. Cases were tested for SARS-CoV-2, influenza and respiratory syncytial virus (RSV), including whole genome sequencing (WGS) on SARS-CoV-2 RNA-positive samples and viral characterisation/sequencing on influenza RNA-positive samples. Descriptive analysis was conducted for SARI cases hospitalised between July 2021 and April 2022.

**Results:**

Overall, we identified 437 SARI cases, the incidence ranged from two to 28 cases per week (0.7–9.2/100,000 hospital catchment population). Of 431 cases tested for SARS-CoV-2 RNA, 226 (52%) were positive. Of 349 (80%) cases tested for influenza and RSV RNA, 15 (4.3%) were positive for influenza and eight (2.3%) for RSV. Using WGS, we identified Delta- and Omicron-dominant periods. The resource-intensive nature of manual clinical data collection, specimen management and laboratory supply shortages for influenza and RSV testing were challenging.

**Conclusion:**

We successfully established SARI surveillance as part of E-SARI-NET. Expansion to additional sentinel sites is planned following formal evaluation of the existing system. SARI surveillance requires multidisciplinary collaboration, automated data collection where possible, and dedicated personnel resources, including for specimen management.

**Key public health message**

**What did you want to address in this study?**

In this study, we wanted to describe how we established sentinel surveillance of severe acute respiratory infection (SARI) in an Irish hospital, to outline the structures, processes and resources that are required for sustainable SARI surveillance, and to bring attention to the public health benefits of SARI surveillance data. We hope that our study helps to guide other countries to establish SARI surveillance.

**What have we learnt from this study?**

Implementing SARI surveillance is challenging. Multidisciplinary collaboration and communication, automating data collection and linkage of datasets where possible are important, as is ensuring that dedicated resources are in place for each step of the surveillance and sample management workflow.

**What are the implications of your findings for public health?**

Surveillance of SARI is important for public health in Ireland. The data are used at hospital and national level to assess the impact and burden of respiratory viruses, to inform public health services planning, and for pandemic preparedness and response. The data are included in a weekly national surveillance report, a weekly European surveillance report, and in European SARI, COVID-19 and influenza vaccine effectiveness monitoring.

## Introduction

With the co-circulation of respiratory viruses, especially during winter, the emergence of severe acute respiratory syndrome coronavirus-2 (SARS-CoV-2) and possibilities for new respiratory pathogens in the future, it is increasingly important to monitor severe acute respiratory infections (SARI) for preparedness and emergency response to national and cross-border public health threats. Population-level SARI surveillance has long been recommended by international agencies [1,2] and in 2020, the COVID-19 pandemic resulted in the European Centre for Disease Prevention and Control (ECDC) accelerating the development of a European SARI surveillance system. This approach combined syndromic surveillance, which collects health data on symptoms of hospitalised patients [3], with results of laboratory testing for pathogens likely to co-circulate in the population, including influenza, SARS-CoV-2 and respiratory syncytial virus (RSV).

Setting up and maintaining a robust SARI surveillance system is challenging and requires multidisciplinary collaboration and considerable resourcing to collect information from various sources [4,5]. In 2020, Ireland had several indicator-based and syndromic surveillance systems to monitor respiratory infections but did not conduct surveillance of hospitalised SARI cases. Our aim was to establish sentinel surveillance of SARI in Ireland as part of the wider European network E-SARI-NET, using funding from ECDC. Specific objectives were to collect SARI data at one pilot hospital site, to conduct temporal analysis of SARI cases, to describe our experiences including the successes and challenges in establishing the surveillance system, and to develop recommendations for SARI surveillance in a network of sentinel Irish hospitals in the future. This work may guide other countries to establish SARI surveillance and contribute to the ongoing development of SARI surveillance.

## Methods

Developing the pilot SARI surveillance system in Ireland was an iterative process; data sources were explored with hospital stakeholders, and data collection and management processes were continually streamlined.

### Setting and study population

The study, initiated by the Health Service Executive (HSE) Health Protection Surveillance Centre (HPSC) in December 2020, was conducted in Saint Vincent’s University Hospital (SVUH), a 484-bed tertiary adult hospital in the Dublin metropolitan area, one of 48 public hospitals in Ireland. The emergency department is the major trauma centre for the South East Dublin region, the hospital catchment area covered 7.5% of the estimated population of Ireland for 2021 (based on the 2016 census of Ireland), a population of 304,145 persons aged 15 years and older. The study population was persons aged 15 years and older admitted to the emergency department.

### Resources

**Figure 1:**
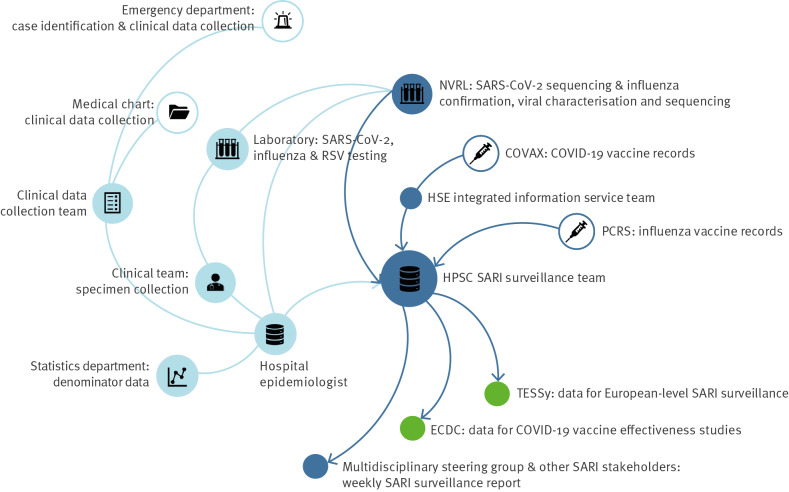
Teams involved in sentinel surveillance of SARI in one hospital, Ireland, 5 July 2021–3 April 2022

COVAX: National COVID-19 Vaccination System; ECDC: European Centre for Disease Prevention and Control; HPSC: Health Protection Surveillance Centre; HSE: Health Service Executive; NVRL: National Virus Reference Laboratory; PCRS: Primary Care Reimbursement Service; SARI: severe acute respiratory infection; TESSy: The European Surveillance System.

A multidisciplinary team was established, comprising teams from key hospital departments (Figure 1).

To address resource challenges caused by high workloads and competing priorities during the COVID-19 pandemic, dedicated SARI surveillance personnel were recruited using funding provided by ECDC to support initial system development. Dedicated personnel included a research nurse for clinical data collection and an epidemiologist co-located between SVUH and HPSC to coordinate laboratory testing and data management.

Guidance was provided to HPSC by ECDC and Epiconcept, who coordinated the European-level project. A specialist in public health medicine and a senior epidemiologist at HPSC coordinated and supervised the project in Ireland, and the pilot project was established by a fellow from the European Programme for Intervention Epidemiology Training (EPIET).

### Data protection

Surveillance was undertaken in accordance with Article 9 of the General Data Protection Regulations (GDPR) 2018 [6], which provides exception to the prohibition of the processing of sensitive personal health information without consent for reasons including protecting against serious cross-border threats to health. Data were collected and stored securely according to a formal data sharing agreement. The HPSC is certified to the information security standard ISO 27001: 2013 and conducts data protection impact assessments (dPIA) to mitigate potential risks in the processing of personal data [7].

### Variables collected

We collected variables in line with the ECDC SARI protocol and included: age, sex, occupation, pregnancy, smoking, employment and healthcare worker status, obesity, COVID-19/influenza vaccination status, symptoms at/before admission, pre-existing chronic conditions, prior hospitalisation, prior SARS-CoV-2 test positivity, hospital and intensive care unit (ICU) admission and discharge dates, medications, respiratory support, complications, outcome, and laboratory test results for SARS-CoV-2, influenza and RSV.

### Data sources

Symptoms, demographics, administrative and clinical data were captured in electronic information systems and/or physical medical charts and required manual data extraction. Hospital laboratory data were extracted in electronic reporting format. Molecular and whole genome sequencing (WGS) data were provided in electronic format by the National Virus Reference Laboratory (NVRL). Records of COVID-19 vaccination were available in electronic format from the National COVID-19 Vaccination System (COVAX) but required data linkage. Ireland does not have an electronic national immunisation system for all vaccines; influenza vaccine records were sourced from the HSE Primary Care Reimbursement Service (PCRS).

Denominator data for the hospital catchment area, provided by the HSE Health Intelligence Unit, were based on geographical boundaries and 2021 population projections estimated by Ireland’s Central Statistics Office [8]. The number of all-cause emergency department admissions was provided weekly by the SVUH statistics department.

**Figure 2:**
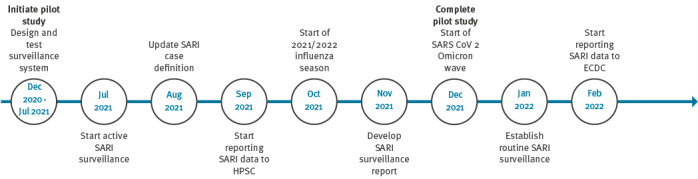
Timeline for implementing sentinel surveillance of SARI in one hospital, Ireland, 2021–2022

ECDC: European Centre for Disease Prevention and Control; HPSC: Health Protection Surveillance Centre; SARI: severe acute respiratory infection.

Active surveillance and data collection commenced in July 2021, 7 months after initiation, during which time the system was designed and field tested. Figure 2 shows the study timeline.

### Case definition

For the first 7 weeks of surveillance, we used the World Health Organization (WHO) SARI case definition to identify cases: a person who had an acute respiratory infection with a history of fever or measured fever of ≥ 38 °C and cough and onset within the last 10 days and who required hospitalisation [9]. During this time, the clinical team observed several patients presenting to the emergency department with symptoms of SARI excluding cough, suggesting that the case definition was not sensitive enough. From 27 August 2021 onwards, we applied a new case definition, partially adapted from ECDC’s clinical criteria for a possible COVID-19 case: a person who had an acute respiratory infection and at least one of the following symptoms: fever, cough, shortness of breath, sudden onset of anosmia (loss of smell), ageusia (loss of taste) or dysgeusia (taste disorder), and onset within the last 14 days and who required hospitalisation (not discharged within 24 h) [10]. Collection of symptom data in disaggregate format facilitated ongoing application of the WHO case definition even after 27 August if required.

Patients whose SARI symptoms were not evident in the emergency department, for example patients with hospital-acquired SARI or patients with SARI who were admitted to SVUH from other hospitals, were not captured in the surveillance system. Deaths recorded were all-cause in-hospital deaths.

## Results

### Surveillance workflow

**Figure 3:**
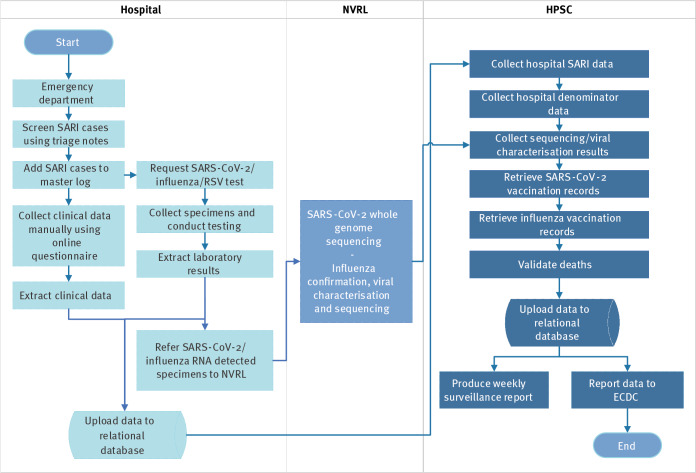
Surveillance workflow for SARI in one hospital, Ireland, 5 July 2021–3 April 2022

ECDC: European Centre for Disease Prevention and Control; HPSC: Health Protection Surveillance Centre; NVRL: National Virus Reference Laboratory; RSV: respiratory syncytial virus; SARI: severe acute respiratory infection.

The surveillance workflow (Figure 3) was as follows:

#### Screening

Patients admitted through the emergency department were screened on weekdays, with weekend admissions screened on Monday. We searched electronic triage notes for keywords; fever, cough, lower respiratory tract infection (LRTI), shortness of breath, pneumonia, COVID-19, influenza and RSV. Anosmia, ageusia and dysgeusia were not included. Those meeting the screening criteria were reviewed clinically.

A daily COVID-19 inpatient list provided by SVUH to medical teams was checked for SARI cases potentially missed at screening. Daily inpatient lists for influenza and RSV were not available, but influenza-positive laboratory records were checked periodically.

We assigned a unique identifying code to the identified cases and added them to a master log using Microsoft Excel software. The master log included the patient’s hospital medical record number (MRN) and was checked for prior SARI admissions to SVUH since the beginning of the study. Collected clinical data were manually entered into an online questionnaire using REDCap (Research Electronic Data Capture), a secure, web-based software platform [11,12].

#### Sample collection and laboratory testing

Molecular investigation for SARS-CoV-2, influenza and RSV RNA was performed for SARI cases using nasopharyngeal samples collected within 48 h of admission, coordinated by the clinical team. Commercial assays were used, including the multiplex GeneXpert Xpress assay (Cepheid, Sunnyvale, United States) for simultaneous SARS-CoV-2, influenza and RSV testing. Molecular results were linked to clinical data from the online questionnaire using Microsoft Access software.

Specimens with a quantification cycle (Cq) value < 25 for SARS-CoV-2 RNA were referred weekly to the NVRL for WGS. Specimens PCR-positive for influenza were referred to the NVRL for confirmation and viral characterisation, those with Cq ≤ 30 were referred for influenza sequencing.

#### Data linkage, analysis and reporting

Weekly reporting of disaggregate data to HPSC commenced in September 2021. SARI hospital records were linked to vaccination and molecular data from sources described previously. Deaths were validated using records of the General Register Office (GRO). As there was no unique patient identifier across all health datasets, we conducted linkage using personal data and matching algorithms. Linkage to COVID-19 vaccination data was established in December 2021 and was conducted retrospectively by the HSE Information Integrated Service Team and validated by HPSC. Data were transformed to the reporting format for The European Surveillance System (TESSy) using R statistical software [13]. A weekly SARI surveillance report was developed in consultation with the multidisciplinary working group and SARI stakeholders. Following successful completion of the pilot in December 2021, routine SARI surveillance commenced.

### Descriptive data analysis

Descriptive analysis was conducted for SARI cases hospitalised between 5 July 2021 and 3 April 2022, which included a 6-month pilot period and 3 months post-pilot period. Data were extracted for analysis on 30 May 2022.

#### Demographic and clinical data

**Figure 4:**
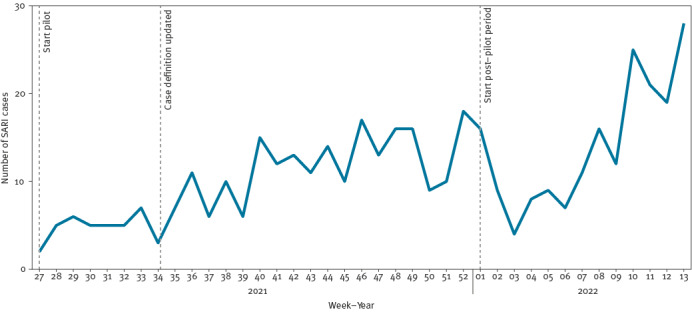
Number of SARI cases by week of admission, one hospital, Ireland, 5 July 2021–3 April 2022 (n = 437)

Overall, 437 SARI cases were identified between 5 July 2021 and 3 April 2022, representing 3.7% (437/11,726) of all-cause admissions to the hospital, via the emergency department. The SARI case numbers ranged from two cases in week 27 of 2021 (week starting 5 July 2021) (0.7/100,000 hospital catchment population) to 28 cases in week 13 of 2022 (week starting 27 March 2022) (9.2/100,000 hospital catchment population) (Figure 4). Only one (0.2%) SARI case, whose symptoms were not recorded in the triage notes, was missed at screening.

SARI: severe acute respiratory infection.

Overall, 229 (52%) cases were male, median age was 71 years (interquartile range (IQR): 53–82 years) (Table). The most common symptoms were cough (n = 375; 86%) and shortness of breath (n = 338; 77%).

**Table:** Demographic and clinical characteristics of SARI cases in one hospital, Ireland, 5 July 2021–3 April 2022 (n = 437)

Characteristics	SARI cases	
	n	%
Sex		
Male	229	52
Female	208	48
Age (years)		
15–24	15	3.4
25–34	18	4.1
35–44	32	7.3
45–54	52	12
55–64	63	14
65–74	74	17
75–84	109	25
≥ 85	74	17
Symptoms		
Cough	375	86
Shortness of breath	338	77
Fever	206	47
General deterioration	160	37
Malaise	106	24
Headache	42	10
Muscular pain	38	8.7
Sore throat	28	6.4
Ageusia	19	4.3
Anosmia	16	3.7
Dysgeusia	9	2.1
Pre-existing chronic conditions		
Hypertension	141	32
Heart disease	137	31
Lung disease	122	28
Cancer	81	19
Neurological disease	71	16
Diabetes	67	15
Asthma	56	13
Kidney disease	39	8.9
Intellectual disability	27	6.2
Immunocompromised	15	3.4
Cystic fibrosis	5	1.1
Complications		
Pneumonia	91	21
Sepsis	13	3.0
Acute respiratory distress syndrome	7	1.6
Myocarditis	2	0.5
Long Covid	2	0.5
Admitted to ICU		
Yes	24	5.5
No	368	84
Unknown	45	10
Respiratory support		
High-flow oxygen therapy (non-invasive ventilation)	214	49
Invasive ventilation	19	4.3
No respiratory support given	148	34
Unknown	56	13
Outcome		
Discharged home	309	71
Still in hospital	55	13
Died in hospital	43	9.8
Discharged to a rehabilitation centre/convalescence home	10	2.3
Transferred to another hospital	9	2.1
Discharged to palliative care	6	1.4
Discharged to a step-down facility	2	0.5
Unknown	3	0.7

ICU: Intensive care unit; SARI: severe acute respiratory infection.

Of 401 cases identified using our updated case definition (between 27 August 2021 and 3 April 2022), 55 (14%) did not have a cough, and 212 (53%) did not have a fever and did not meet the WHO SARI case definition. Most of those without cough (n = 32; 58%) had SARS-CoV-2 RNA detected, four (7.3%) had influenza RNA detected, and none had RSV RNA detected. Ninety-four (44%) of those without fever had SARS-CoV-2 RNA detected, eight (3.8%) had influenza RNA detected, and four (1.9%) had RSV RNA detected.

The vast majority (n = 381; 87%) of the 437 cases had at least one pre-existing chronic condition; most commonly hypertension (n = 141; 32%) and heart disease (n = 137; 31%), while 91 (21%) developed pneumonia. Respiratory support was provided to 233 (53%) cases, 19 (4.3%) cases received invasive ventilation. Twenty-four (5.5%) cases had at least one ICU stay. At the time of data extraction, 336 (77%) cases had been discharged home or to other facilities (Table), 55 (13%) were still in hospital, 43 (9.8%) died in hospital, and the outcome was not reported for three (0.7%). The median hospital length of stay (LOS) was 5 days (IQR: 3–11) for cases discharged home, 14 days (IQR: 7–20) for cases transferred to another facility, 13 days (IQR: 5–25) for cases who died in hospital, and 68 days (IQR: 61–82) up to the date of data extraction for cases still in hospital.

### Laboratory testing results

Overall, 431 (99%) cases were tested for SARS-CoV-2 RNA and six (1.4%) were not tested. Of those tested, 226 (52%) had SARS-CoV-2 RNA detected, 190 (44%) had not, and 15 (3.5%) were classified as low-level SARS-CoV-2 RNA detected (Cq ≥ 30). In total, 349 (80%) cases were tested for influenza and RSV RNA and 88 (20%) were not tested (for reasons outlined below). Of those tested, 15 (4.3%) had influenza A detected (13 were influenza A(H3) and two were not subtyped), influenza B was not detected in any specimen, and eight (2.3%) had RSV RNA detected.

**Figure 5:**
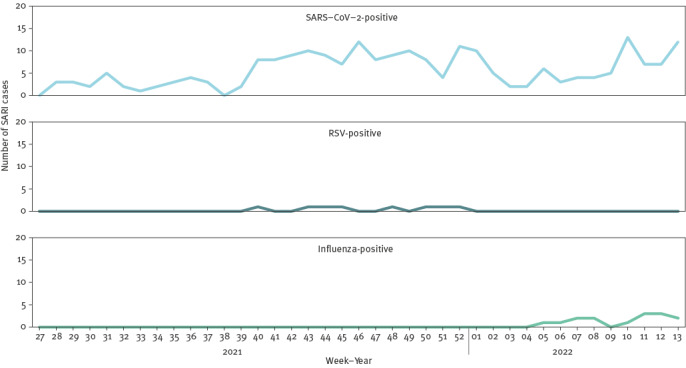
Number of SARI cases with SARS-CoV-2 (n = 226), RSV (n = 8) or influenza (n = 14) RNA detected, by week of admission, one hospital, Ireland, 5 July 2021–3 April 2022

RSV: respiratory syncytial virus; SARI: severe acute respiratory infection; SARS-CoV-2: severe acute respiratory syndrome coronavirus 2.

Two (0.6%) influenza-positive SARI cases were co-infected with SARS-CoV-2, and one (0.3%) RSV-positive case was co-infected with SARS-CoV-2. Of 349 cases tested for all three pathogens, 145 (42%) were negative for all three. Trends in laboratory results are shown in Figure 5.

**Figure 6:**
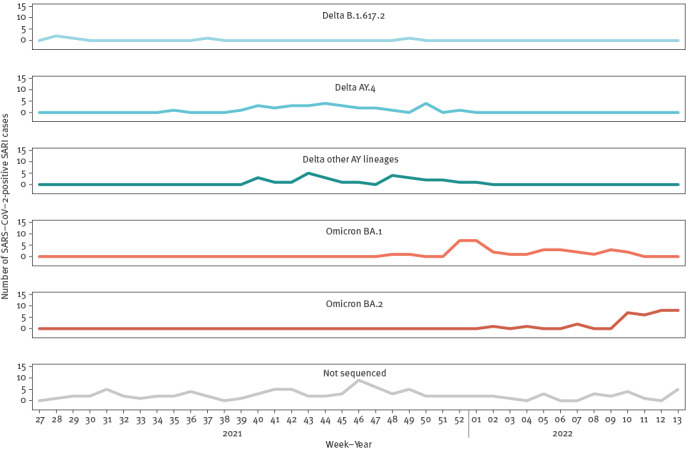
Pango lineage of SARI cases with SARS-CoV-2 detected (n = 226) by week of admission, one hospital, Ireland, 5 July 2021–3 April 2022

SARI: severe acute respiratory infection; SARS-CoV-2: severe acute respiratory syndrome coronavirus 2.

We conducted WGS for 130 (58%) SARI cases with SARS-CoV-2 RNA detected at an adequate viral load. Overall, 63 (48%) were Delta variants (Phylogenetic Assignment of Named Global Outbreak Lineages (Pangolin) designation B.1.617.2 and AY lineages) and 67 (52%) were Omicron variants (BA.1 and BA.2 lineages) (Figure 6).

Twelve influenza A(H3)-positive samples were genetically characterised, all of which clustered in the A(H3) genetic clade subgroup 3C.2a1b.2a.2, represented by the A/Bangladesh/4005/2020 virus, the predominant subgroup circulating globally during the 2021/22 season. The A/Bangladesh viruses were antigenically diverse from the A(H3)/Cambodia/e0826360/2020 vaccine strain included in the northern hemisphere 2021/22 vaccine [14].

### Challenges

There were multiple challenges in establishing SARI surveillance, these are outlined in the Box.

Box **Challenges in establishing sentinel SARI surveillance in one hospital, Ireland, 5 July 2021–3 April 2022**

**Clinical data collection**

- Collection of clinical data was resource-intensive because medical charts were located on restricted access COVID-19 wards or were in use by other hospital teams.
- Personnel resources were insufficient during peak periods including the beginning of the COVID-19 Omicron pandemic wave, which started in Ireland on 19 December 2021, and during periods of annual leave.

**Specimen collection and laboratory testing**

**SARS-CoV-2:**

- Specimens were not collected in hospital for a total of 21 (4.8%) SARI cases. Nine cases had a short hospital length of stay and were discharged before specimens could be collected, reasons why specimens were not collected were unknown for 12. Results of SARS-CoV-2 RNA testing carried out shortly before hospital admission were obtained for 15 of the 21.
- SARS-CoV-2 WGS could not be conducted for 96 (42%) of the 226 cases with SARS-CoV-2 RNA detected. The Cq values were too high for 52 (23%) and sequences could not be generated for a further four (1.8%).
- Fourteen (6.2%) specimens were disposed in error before WGS referral, and four (1.8%) were referred to the wrong laboratory.
- Other issues preventing WGS included the unavailability of specimens for cases investigated for SARS-CoV-2 RNA before hospital admission (n = 15; 6.6%), and insufficient specimen volume sometimes relating to routine testing for variants of concern (VOC) (n = 7; 3.1%) which ceased for all SARI specimens in September 2021.

**Influenza and RSV:**

- Forty-one (9.4%) SARI cases hospitalised before the start of the 2021/22 influenza season, which started in Ireland on 4 October 2021, could not be tested for influenza and RSV because of laboratory supply issues for multiplex kits.
- For those admitted to SVUH under the COVID-19 care pathway, the request for influenza and RSV testing was not added at the time of sampling but was later added by the SARI team. The laboratory policy to dispose SARS-CoV-2 RNA-negative specimens after 1 week meant that specimens could not be retrieved at a later date for testing, this resulted in disposal of specimens for 19 (4.3%) cases before influenza and RSV testing could be conducted.
- Specimens for three (0.7%) cases were referred for SARS-CoV-2 WGS before influenza and RSV testing could be conducted.
- Influenza and RSV analytical quality control failures occurred for the specimens of four (0.9%) cases.
- Specimens were not collected for the remaining 21 (4.8%) cases as previously mentioned.

Cq: quantification cycle; RSV: respiratory syncytial virus; SARI: severe acute respiratory infection; SARS-CoV-2: severe acute respiratory syndrome coronavirus 2; WGS: whole genome sequencing.

## Discussion

We successfully established a SARI surveillance system in Ireland, using routine emergency department records to identify cases. Contributing factors included, but were not limited to, multidisciplinary collaboration, having a dedicated SARI epidemiologist co-located between the hospital site and HPSC, guidance provided by Epiconcept, which included development of a SARI protocol, and ECDC support including funding allocated to recruit dedicated personnel.

Active daily screening and detailed review of clinical records was a strength of the surveillance system, in contrast to passive register-based surveillance in which there may be uncertainties around case classification and where laboratory diagnoses are frequently unavailable [15]. Active surveillance systems provide the most timely and accurate information, however, passive register-based systems are sometimes more attractive as they require less personnel resources [16,17]. We explored the possibility to establish a register-based system using the International Statistical Classification of Diseases 10th Revision (ICD-10) codes collected as part of the Hospital Inpatient Enquiry Scheme (HIPE) in Ireland, but validated data were unavailable until at least 6 months after the end of the previous year and the time lag to acquisition of validated data was considered too long [18].

The collected data demonstrate that syndromic SARI surveillance can be used to monitor SARI incidence and trends and to provide insights into the burden of respiratory disease arising from detected respiratory infections. We updated our case definition during the study, from the WHO SARI case definition that was developed in 2014 for influenza surveillance [9] to a case definition which we partially adapted from ECDC’s clinical criteria for a possible COVID-19 case [10]. Over half of the 401 cases identified after we updated our case definition did not meet the WHO 2014 case definition as they did not have a fever (n = 212; 53%) or a cough (n = 55; 14%). A high proportion of these cases had SARS-CoV-2 RNA detected (44% and 58%, respectively), highlighting potential limitations of the WHO case definition for use in SARI surveillance in the present-day. A detailed evaluation of the performance of our case definition against the WHO SARI case definition and other different SARI case definitions that may be in use at the national level in other countries would be beneficial.

Most SARI cases were tested for SARS-CoV-2 and for influenza and RSV (99% and 80%, respectively). Laboratory supply issues for influenza and RSV testing before the beginning of the influenza season highlighted the need for laboratories involved in SARI surveillance to have access to supplies through the health service central procurement process throughout the year, even when these pathogens are not widely circulating in the population.

Surveillance of SARI provides a different measure of disease activity than national indicator-based and syndromic surveillance systems to monitor respiratory infections. All systems are fundamental to comprehensive surveillance of respiratory diseases, each have different goals and there are some overlaps. Influenza positivity among our SARI cases reflected low influenza activity overall during the 2021/22 influenza season in Ireland and an increase in influenza A virus circulation towards the end of the study period [19]. The rate of influenza-like illness (ILI) GP phone consultations reported in Ireland for the same period peaked earlier, in week 1 of 2022, which was likely due to respiratory pathogens other than influenza circulating in the population at that time [19]. The low number of SARI cases that tested positive for RSV throughout the study period was probably due to the lack of RSV testing for SARI cases (resulting from supply issues with multiplex kits between July and October 2021) during a period of high RSV activity in Ireland and also reflected the age profile of the patient cohort at the SARI hospital site (≥ 15 years) [19]. We did not compare national trends in laboratory-confirmed COVID-19 with trends in SARS-CoV-2 positivity from SARI surveillance because the national COVID-19 testing policy changed in early 2022 and because of the delay in COVID-19 notifications in December 2021 and January 2022 which occurred due to increased demands on public health services during the beginning of the Omicron wave [20]. Whole genome sequencing identified Delta and Omicron SARS-CoV-2 variants. The first Omicron variant was identified in a SARI case admitted to SVUH on 1 December 2021 (week 48 of 2021), only 6 days after the specimen collection date of the first Omicron case notified in Ireland [21]. These data demonstrate that sentinel surveillance of SARI could be used to transition from emergency COVID-19 surveillance to objective-driven surveillance with integrated surveillance of respiratory viruses and genomic surveillance of representative samples, as recommended by ECDC in 2021 [22]. The SARI surveillance also provides flexibility to add other or novel respiratory pathogens circulating in the population in the future.

Development of the surveillance system was not without challenges. Manual clinical data collection was resource-intensive and sensitive to elements beyond the control of the surveillance team, including difficulties obtaining medical charts. To reduce this burden, chart retrieval frequency was reduced to twice per case; once following admission and once following discharge, with variables relating to severity (ICU admission, respiratory support, complications, treatment and outcome) collected after discharge only. A consequence of delayed data collection for indicators of severity was that they could not be included in trend analyses as they would not reflect the true situation. Resource challenges were heightened during peak periods of virus circulation, particularly the COVID-19 Omicron wave which began in Ireland in December 2021, requiring an additional resource to be recruited to assist with clinical data collection. To achieve sustainable surveillance, clinical data collection should be automated where possible. In Ireland, hospitals have different data infrastructures and methods for storing health data. Hospital information systems should be standardised nationally where possible in the future, and developments including electronic medical records should ensure capability for syndromic surveillance (not limited to SARI). Furthermore, the absence of a unique personal identifier across health datasets resulted in surveillance inefficiencies. Individual health identifiers are a fundamental enabler for such surveillance projects and should be prioritised in Ireland [23]. Specimen management issues were particularly prominent during critically busy times in the laboratory including during the Omicron wave, when frozen storage space was exceeded and ambient specimen storage and retrieval could not be managed efficiently. Lack of dedicated personnel for specimen management resulted in disposal or referral of specimens before influenza or RSV testing could be conducted, highlighting the need for dedicated personnel to manage SARI samples promptly and for laboratory personnel to be aware that testing needs to be done for both clinical and surveillance purposes. Sample management needs are frequently underestimated in infectious disease surveillance; however, personnel resourcing is challenging given severe recruitment and retention difficulties in medical laboratories in Ireland’s hospitals [24].

Despite the fact that SARI surveillance currently exists in only one sentinel hospital site, it has proved to be important to public health in Ireland. The SARI data are used at hospital level to assess the impact and burden of respiratory viruses, and at the national level to inform public messaging and planning for winter health services. A surveillance report is published weekly in Ireland [25], and disaggregate data are reported to ECDC (via TESSy) and are included in the weekly bulletin Flu News Europe [26] and European SARI COVID-19 vaccine effectiveness studies [27]. Further, SARI surveillance is an important component of pandemic preparedness and planning, providing a useful distinction between infection and morbidity to inform the pandemic response.

We recommend expansion of SARI surveillance to additional sentinel sites in Ireland at the earliest opportunity, to benefit from the strengthened relationships that followed increased cross-sectoral and intra-sectoral work during the COVID-19 pandemic and from increased interest and perceived need expressed by clinicians in other hospitals for the SARI surveillance data. Benefits of SARI data to patient care could be explored to support integration of surveillance into clinical settings [5]. Before expanding SARI surveillance in Ireland, a formal evaluation should be conducted, to appraise the sensitivity of the surveillance system, the potential for missed cases, and the quality, efficiency and usefulness of the data collected.

The main limitation of our study was that SARI surveillance was limited to one adult hospital and was not nationally representative. Considerations for future SARI hospital site selection include a paediatric hospital and sentinel site hospitals in other regions. Viral respiratory infections are a frequent cause of hospitalisations in young children, with a considerable proportion caused by RSV and influenza each winter [28–30]. Other considerations include, but are not limited to, hospital data infrastructure, use of electronic medical records, resourcing needs for all steps of the surveillance workflow, hospital type, size, speciality and geographical location, deprivation level and other aspects of the hospital catchment area.

## Conclusions

We successfully established sentinel surveillance of SARI in an Irish hospital as part of a wider European network, with integrated testing for SARS-CoV-2, influenza and RSV RNA. The SARI surveillance data have proved to be important to public health in Ireland, and we recommend that surveillance be expanded to additional sentinel sites at the earliest opportunity, following formal evaluation of the existing system. We re-iterate the importance of unique health identifiers across all health datasets to enable data linkages and recommend implementation as a priority in Ireland. Future scale-up should ensure that SARI data collection be automated where possible and personnel resourcing requirements should not be underestimated, including for sample management.
